# Bullous Diseases in the Perioperative Setting: Anesthetic Considerations

**DOI:** 10.7759/cureus.101515

**Published:** 2026-01-14

**Authors:** Sarah A Bokaee, Akhil Patel

**Affiliations:** 1 Department of Anesthesiology and Critical Care Medicine, George Washington University School of Medicine and Health Sciences, Washington, DC, USA

**Keywords:** airway management, anesthesia management, bullous pemphigoid, bullous skin disease, dystrophic epidermolysis bullosa, pemphigus vulgaris, perioperative medicine

## Abstract

Bullous skin disorders, including epidermolysis bullosa, pemphigus, and bullous pemphigoid, pose distinctive anesthetic challenges due to extreme cutaneous fragility, mucosal involvement, fluid imbalances, an increased risk of infection, and multisystem comorbidities. Although these patients frequently require surgical or procedural care, evidence guiding perioperative management is limited to isolated case reports and small series. This systematic review aimed to synthesize contemporary data on anesthetic and perioperative strategies for patients with autoimmune and inherited bullous diseases. We conducted a PROSPERO-registered review (CRD420251160409) of English-language studies published from 2015 to 2025 that reported perioperative management of patients with bullous skin disorders undergoing any form of anesthesia. A total of 26 studies met the inclusion criteria. Across these reports, we identified 17 distinct intraoperative precautions, most of which focused on minimizing shear, pressure, and adhesive trauma. The most frequently described interventions included padded or lubricated pulse oximetry, ocular lubrication, cotton interfaces under blood pressure cuffs, non-adhesive ECG monitoring, and extensive pressure-point padding. Airway-specific strategies, such as lubrication of equipment, smaller endotracheal tubes, and tape-free fixation, were consistently recommended to mitigate risks associated with mucosal erosions, bleeding, and airway narrowing. Less common measures addressed environmental warming, humidification, vascular access using ultrasound, and preoperative fluid or electrolyte optimization. The predominance of skin- and mucosa-directed precautions reflects the central role of dermatologic vulnerability in perioperative planning. However, reviewed studies also emphasized broader systemic considerations, including steroid-related metabolic changes, cardiovascular and neurologic comorbidities, renal impairment, infection risk, and the need for multidisciplinary coordination. Despite consistent themes, no standardized guidelines exist regarding preferred anesthetic techniques, agent selection, or postoperative monitoring. In conclusion, current evidence underscores the importance of atraumatic handling, meticulous airway planning, and individualized, multisystem assessment in the anesthetic care of patients with bullous diseases. Future prospective studies are needed to establish evidence-based protocols and optimize perioperative outcomes in this high-risk population.

## Introduction and background

Bullous diseases, such as epidermolysis bullosa, pemphigus, and bullous pemphigoid, are rare conditions that pose unique anesthetic and perioperative challenges due to fragile skin and mucosal involvement. Existing literature consists mostly of case reports and small series, but there is no comprehensive synthesis of anesthetic strategies or perioperative outcomes for this patient population. This review aims to systematically collate and summarize current evidence, identify best practices, and highlight gaps in knowledge to guide anesthesiologists in safely delivering anesthetics to patients with bullous diseases.

Review objectives

The primary objective of this review is to systematically identify and synthesize the literature on anesthetic and perioperative management in patients with bullous diseases, including describing perioperative approaches used in patients with bullous skin disorders and summarizing airway management considerations in those with mucosal or skin involvement.

Bullous diseases are autoimmune blistering disorders characterized by autoantibody-mediated disruption of epidermal adhesion. In pemphigus, pathogenic IgG autoantibodies target desmogleins 1 and 3 within desmosomes, leading to intraepidermal blistering. In contrast, bullous pemphigoid and epidermolysis bullosa acquisita involve antibodies against hemidesmosomal proteins BP180, BP230, and type VII collagen, causing subepidermal separation and dermal inflammation, particularly when lateral shearing forces are applied to the skin. Complement activation, eosinophilic infiltration, and cytokine-driven tissue injury contribute to chronic mucocutaneous fragility and systemic immune activation [1].

Although primarily cutaneous, these autoimmune processes have widespread systemic consequences. Manifestations of epidermolysis bullosa dystrophica include severe scarring with fusion of the digits (pseudosyndactyly), constriction of the oral aperture (microstomia), esophageal stricture, and dysplastic teeth. Malnutrition, anemia, fluid loss, electrolyte derangements, and hypoalbuminemia are common, most likely reflecting chronic infection, debilitation, and renal dysfunction. Survival beyond the second decade is unusual.

Furthermore, cross-reactivity between epithelial and neuronal antigens, along with chronic systemic inflammation, underlies the strong association between bullous pemphigoid and neurologic diseases such as dementia, Parkinson’s disease, and stroke [2,3]. Long-term corticosteroid and immunosuppressive therapy further contributes to cardiovascular, metabolic, and musculoskeletal complications [4-6]. Chronic inflammation promotes endothelial dysfunction and atherosclerosis, while steroid-induced hyperglycemia and bone demineralization exacerbate perioperative risk [7,8]. Renal dysfunction may arise from immune complex deposition or nephrotoxic medications [1,9].

Mucosal involvement is particularly relevant to anesthesia and perioperative care. Bullae and erosions of the oropharynx, larynx, esophagus, and trachea can cause airway narrowing, bleeding, and stenosis, predisposing to difficult intubation and postoperative edema [10,11]. Esophageal strictures and erosions may also increase aspiration risk, and extensive oropharyngeal involvement makes eating painful. Patients may decrease oral intake to the point of malnutrition. The risk of secondary infection is substantial.

Despite these multisystem implications, anesthetic management for patients with bullous diseases remains poorly defined. Most published data consist of case reports or small series describing individualized approaches to airway protection, analgesia, and positioning. There are no standardized guidelines or recommendations addressing preferred anesthetic technique, agent selection, or postoperative monitoring in this population. Accordingly, this systematic review aims to synthesize existing literature on perioperative and anesthetic management in patients with autoimmune bullous diseases to inform multidisciplinary best practices.

## Review

Materials and methods

Our review was prospectively registered in PROSPERO (ID: CRD420251160409). We included studies reporting on patients with bullous skin diseases (epidermolysis bullosa (junctional, simplex, dystrophic, or unspecified), pemphigus vulgaris, pemphigus foliaceus, bullous pemphigoid, and epidermolysis bullosa acquisita) who underwent any form of perioperative or procedural anesthesia, including general, regional, local, or procedural sedation. Eligible study designs included case reports, case series, retrospective observational studies, and CME reports containing relevant primary patient data, found using the literature search strategy outlined in Table 1. Only studies published in English from January 2015 to October 2025 were included to capture contemporary anesthetic practices. Studies were excluded if they involved non-bullous dermatologic conditions, animal subjects, or if they did not use anesthesia.

**Table 1:** Literature search strategy.

Concept	Search Strategy
Bullous diseases	“Pemphigus”[Mesh] OR pemphigus[tiab] OR “Pemphigoid, Bullous”[Mesh] OR “bullous pemphigoid”[tiab] OR “Pemphigoid, Benign Mucous Membrane”[Mesh] OR “cicatricial pemphigoid”[tiab] OR “mucous membrane pemphigoid”[tiab] OR “Epidermolysis Bullosa”[Mesh] OR “epidermolysis bullosa”[tiab]
Anesthesia-related terms	“Anesthesia”[Mesh] OR anesthesia[tiab] OR anaesthesia[tiab] OR anesthetic[tiab] OR anaesthetic[tiab] OR “Anesthesiology”[Mesh] OR anesthesiology[tiab]
Boolean combination	Bullous diseases terms AND anesthesia-related terms

Results

**Figure 1:**
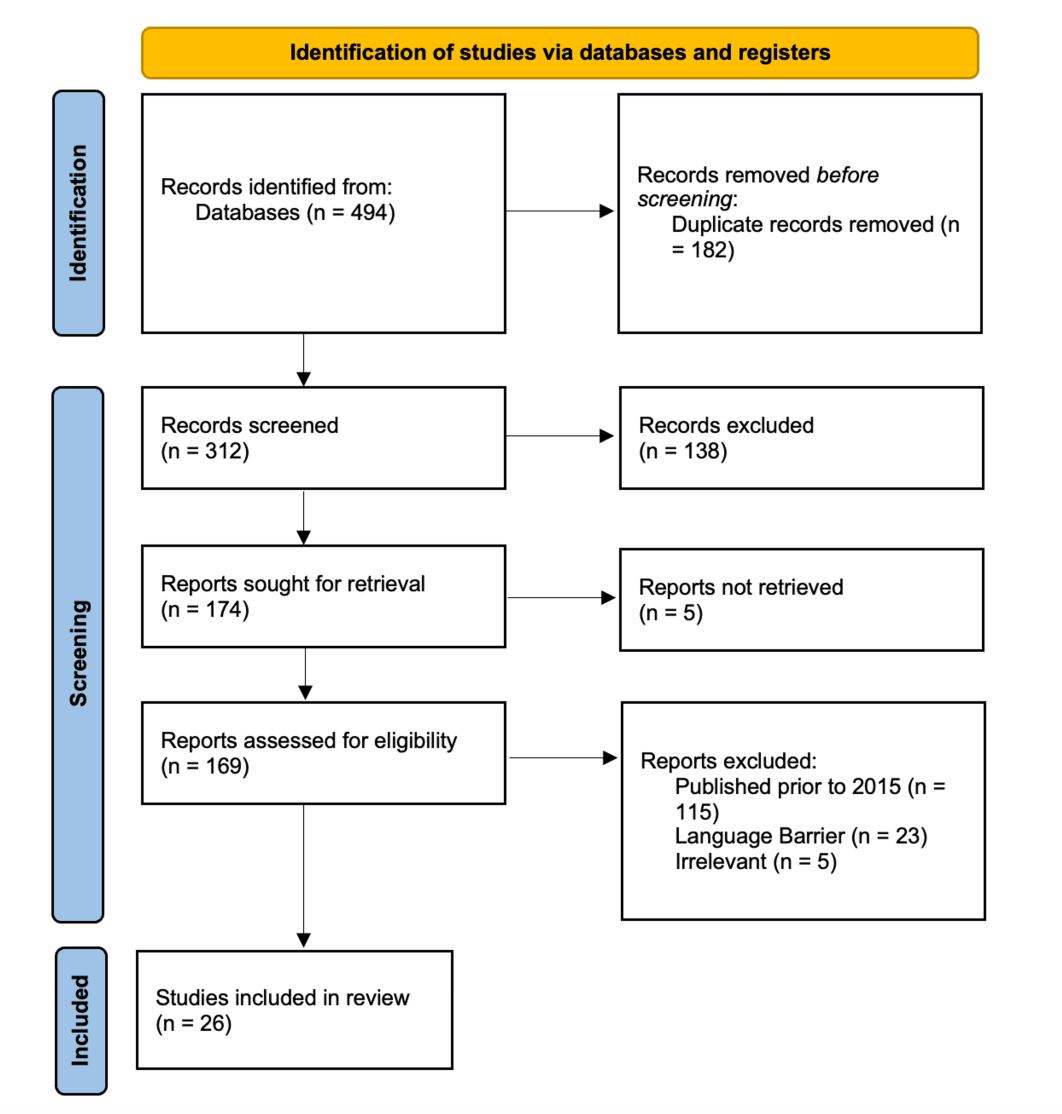
PRISMA flow diagram. PRISMA: Preferred Reporting Items for Systematic Reviews and Meta-Analyses

As noted in the PRISMA (Preferred Reporting Items for Systematic Reviews and Meta-Analyses) flow diagram in Figure 1, a total of 494 records were identified through database searching (PubMed, Cochrane, and Scopus) and additional sources. After removing duplicates, 312 unique records remained for title and abstract screening. Of these, 138 were excluded, leaving 174 articles for full-text assessment. Following full-text review, 148 articles were excluded for reasons including publication before 2015 (n=115), article unavailable (n=5), duplicate or wrong population (n=2), not perioperative/anesthesia related (n=2), not focused on bullous disease (n=1), insufficient data or language barrier (n=23), and other reasons. In total, 26 studies met the inclusion criteria.

A total of 15 distinct intraoperative precautions were identified across the reviewed studies in Table 2, reflecting strategies to minimize cutaneous and mucosal trauma in patients with bullous dermatoses undergoing anesthesia. The most frequently reported interventions focused on non-invasive monitoring and prevention of mechanical injury to fragile skin.

**Table 2:** The 26 studies used in this systematic review of bullous diseases and anesthetic implications to be aware of. For each report listed above, the recommended precautionary measure from the corresponding review is listed.

Citation	Study Type	Disease Type	Number of Patients	Airway	Anesthesia	Precautions Taken	Outcome
Aikawa et al. [12]	Case report	Epidermolysis bullosa (dystrophic)	1	Limited mouth opening and severe adhesion in the oral cavity.	Regional (ultrasound-guided transversus abdominis plane block (TAPB))	GelECG; NSM	No complications
Al-Abadi A et al. [13]	Case report	Epidermolysis bullosa (dystrophic)	1	Ankyloglossia and a smooth tongue with obliteration of the buccal and lingual vestibules.	General	PeriOpt	Forehead/lip/oral mucosa sloughing; healed with minimal scarring; anemia treated with iron
Araújo, et al. [14]	Case report	Epidermolysis bullosa (dystrophic)	1	Limited mouth opening due to scarring and contractures at the corners of the mouth, adherent tongue to the floor of the mouth.	Spinal	ClipOx; NIBPcotton; GelECG; EyeLub; PressPad; FoamOT	No complication
Arikrishnan et al. [15]	Case report	Epidermolysis bullosa (dystrophic)	1	Multiple palatal and buccal mucosal erosions, active bleeding ulcer on the dorsum of the tongue, multiple hypoplastic teeth, external appearance of a difficult airway.	Regional (caudal block)	ClipOx; EyeLub; PressPad	No complication
Bowen L et al. [16]	CME article	Epidermolysis bullosa (non-specified)	N/A	N/A	Multimodal Preferred	ClipOx; NIBPcotton; GelECG; EyeLub; LubAirway; NoTapeETT	No complication
Brooks et al. [17]	Retrospective review	Epidermolysis bullosa (non-specified)	37	Most had airway bullae.	Mixed	NSM; MultiCare	New skin/mucosal injury 4%; desaturation 21%; some blood/iron transfusions
Chen et al. [18]	Case report	Epidermolysis bullosa (simplex)	1	N/A	General	ClipOx; NIBPcotton; GelECG; EyeLub; PressPad; FoamOT; NoAdh	Small bullae 2 cm from the adhesive drape
Chia et al. [19]	Case report	Epidermolysis bullosa (dystrophic)	1	Airway swelling and perceived risk of bullae formation, an otolaryngologist was consulted.	General	ClipOx; EyeLub; NoAdh; PressPad	Post-operative nausea and vomiting (day 1)
Fitzmaurice et al. [20]	Case report	Epidermolysis bullosa (dystrophic)	1	Severely restricted mandibular protrusion, microstomia, a ﬁxed tonguesecondary to scarring. The soft tissues were extremely friable, resulting in obscured visibility. Fiberoptic visualization of the vocal cords was unsuccessful.	Genearl	ClipOx; NIBPcotton; GelECG; EyeLub; NoAdh; LubAirway; NoTapeETT	No complications
Froyshteter et al. [21]	Case report	Epidermolysis bullosa (simplex)	1	N/A	Spinal	ClipOx; NIBPcotton; GelECG; EyeLub; PressPad; WarmEnv; LubAirway	No complications
Gafsi et al. [22]	Case report	Epidermolysis bullosa (dystrophic)	2	Very limited mouth opening, buccopharyngeal lesions, an adherent tongue, a thyro-mental distance of 50 mm at maximum, difficult subluxation of the jaw.	General	NoAdh; SiT; PressPad; FoamOT	Generalized scar lesions, pustules, joint contractures; pruritus
Inoue et al. [23]	Case report	Epidermolysis bullosa (dystrophic)	1	Mouth could open only up to approximately 5 mm, rampant inflammation.	Regional (femoral nerve block)	ClipOx; NIBPcotton; EyeLub; LubAirway; NoTapeETT	Delayed surgical wound healing; no new blisters
Kiss et al. [24]	Case report	Epidermolysis bullosa (simplex)	1	N/A	General	SiT; NoAdh	No complication
Mello et al. [25]	Case report	Epidermolysis bullosa (dystrophic)	1	Ulcerations and bullous lesions on the interior of the mouth.	General	ClipOx; NIBPcotton; GelECG; EyeLub; NoAdh; NoTapeETT	Intraoral blister formation
Mishra et al. [26]	Case report	Epidermolysis bullosa (dystrophic)	1	Restricted mouth opening, tapered narrowing (short segment) of proximal esophagus, suggestive of either an esophageal stricture. Gelatinous mucosa of the esophagus with easy bruising proximal to the stricture site.	General	ClipOx; NIBPcotton; GelECG; EyeLub; PressPad; FoamOT; NoAdh	No complication
Mittal et al. [9]	Review article	Epidermolysis bullosa (non-specified)	N/A	Progressive airway deterioration is common.	General, Regional, Neuraxial are safe.	ClipOx; NIBPcotton; GelECG; EyeLub; PressPad	N/A
Mummert et al. [27]	Case report	Epidermolysis bullosa (junctional)	1	N/A	General	NoTapeETT; EyeLub; LubAirway)	Minimal blistering from intubation
Narejo et al. [28]	Case report	Epidermolysis bullosa (dystrophic)	1	Hoarseness of voice, restricted mouth opening due to contractures at the angle of the mouth, and denuded oral mucosa.	General	LubAirway; NoTapeETT	No complication
Ng et al. [29]	Case report	Epidermolysis bullosa (dystrophic)	1	Limited mouth opening, minimal interincisor distance due to the growth of permanent incisors, and restricted neck movement from scarring.	General	ClipOx; NIBPcotton; GelECG; EyeLub; PressPad; LubAirway	No complication
Noda et al. [30]	Case report	Epidermolysis bullosa (dystrophic)	1	Several erosions.	General	ClipOx; NIBPcotton; GelECG; EyeLub; PressPad; WarmEnv; PeriOpt	No complication
Ozer et al. [31]	Case report	Epidermolysis bullosa (non-specified)	1	Scar formation on the face and neck that restricted movement.	General	ClipOx; NIBPcotton; GelECG; EyeLub; PressPad; NoAdh	ICU for anemia/tachycardia/transfusion reaction
Özkan et al. [32]	Case report	Epidermolysis bullosa (non-specified)	1	Inside of the mouth was bleeding and had a fragile structure.	General	ClipOx; NIBPcotton; GelECG; EyeLub; PressPad; BloodTx	Fatal outcome
Salviz et al. [33]	Case report	Epidermolysis bullosa (non-specified)	1	Progressive scarring into the oropharynx and esophagus, patient had restricted mouth opening.	Regional (brachial plexus block)	ClipOx; NIBPcotton; GelECG; EyeLub; PressPad; BloodTx	No complication
Singh et al. [34]	Case report	Epidermolysis bullosa (dystrophic)	1	Tongue attachment to the floor of the mouth with involvement of the cheek and perioral area, involvement of the pharynx or trachea could not be visually confirmed.	General	ClipOx; NIBPcotton; GelECG; EyeLub; PressPad; BloodTx	Post-op analgesia with IV paracetamol
van den Heuvel et al. [35]	Retrospective review	Epidermolysis bullosa (dystrophic)	9	Airway problems were "very frequent"	Regional (median nerve block)	ClipOx; NIBPcotton; GelECG; EyeLub; PressPad; WarmEnv; PeriOpt; BloodTx	No complication
Yukawa et al. [36]	Case report	Epidermolysis bullosa (simplex)	1	N/A	General	BloodTx; WarmEnv; NoAdh; NoTapeETT; EyeLub	No complication

Table 3 summarizes the types of each precautionary measure identified across studies. The majority of interventions (approximately 79%) addressed cutaneous or mucosal protection, including padding, lubrication, minimization of shear forces, and avoidance of adhesives. Airway-related modifications accounted for approximately 11% of reported measures, whereas systemic and environmental precautions accounted for the remaining 10%, including transfusion or nutritional optimization and maintenance of thermal or humidified conditions.

**Table 3:** Recommended precautionary measures and their respective frequencies in the 26 studies included in the review.

Short-Hand	Precaution Taken	Frequency in Reviews
ClipOx	clip-on/earlobe oximeter (nonadhesive)	18
EyeLub	ophthalmic ointment, lubricated gauze/goggles	20
GelECG	modified ECG electrodes (gel or reduced adhesive)	16
NIBPcotton	cotton padding under BP cuff	16
PressPad	gauze/foam padding at pressure points	15
NoAdh	no adhesives; use non-adhesive dressings/barrier	9
LubAirway	lubricated airway equipment (mask, tube, nasal cannula)	7
NoTapeETT	ETT secured without adhesive (fastener, gauze, sutures)	7
NSM	no sliding movements (avoid shear)	2
FoamOT	foam mattress/egg-crate padding on OR table	4
BloodTx	perioperative transfusion management	6
WarmEnv	warmed OR, humidified O₂, blanket	4
PeriOpt	perioperative optimization (nutrition, fluids, Hb correction)	4
SiT	silicone-based tape/dressing (Mepilex, Mepitac, Mepitel)	3
MultiCare	multidisciplinary approach	1

Across the 26 included studies, several consistent precaution categories emerged, reflecting a shared goal of minimizing skin and mucosal trauma during anesthesia. Lubrication-based strategies were the most frequently reported, including ocular protection with lubricating ointments or moistened gauze (EyeLub, n=20), lubricated airway equipment such as facemasks and endotracheal tubes (LubAirway, n=7), and petroleum- or gel-based interfaces for monitoring devices. Avoidance of adhesives was another major theme, including modified ECG electrodes with gel or reduced adhesive (GelECG, n=16), non-adhesive pulse oximetry techniques (ClipOx, n=18), use of silicone-based dressings (SiT, n=3), and complete avoidance of tapes or adhesive-based fixation when possible (NoAdh, n=9). Many studies additionally emphasized avoiding shear forces, including explicit recommendations to prevent sliding or repositioning after draping (NSM, n=2) and to maintain adequate lubrication and protective interfaces to reduce friction.

A second major cluster of interventions centered on padding and pressure redistribution, including cotton interfaces under non-invasive blood pressure cuffs (NIBPcotton, n=16), extensive padding of bony prominences and pressure points (PressPad, n=15), and use of foam or egg-crate operating table mattresses (FoamOT, n=4). Systemic perioperative considerations were mentioned less frequently but remained relevant: several studies described perioperative optimization, such as correction of anemia, hydration, and nutritional support (PeriOpt, n=4), as well as transfusion planning for severe disease or anticipated blood loss (BloodTx, n=6). Environmental warming or humidified oxygen (WarmEnv, n=4) appeared occasionally as supportive measures to maintain mucocutaneous moisture and prevent skin cracking.

Notably, while skin protection dominated the literature, only one study explicitly advocated a multidisciplinary care approach (MultiCare, n=1). Given the complex dermatologic, immunologic, airway, and hemodynamic issues encountered in bullous disease, the rarity of this recommendation highlights an important opportunity to expand coordinated perioperative care models.

Taken together, these findings illustrate a broad but consistent pattern of preventive strategies emphasizing skin integrity preservation, non-adhesive monitoring, and mucosal lubrication. The predominance of simple measures, such as padding and lubrication, highlights the feasibility of dermatology-informed anesthesia practices. Furthermore, these findings highlight the need for structured, systems-based approaches to anesthetic care in bullous diseases in order to reduce iatrogenic trauma and optimize perioperative outcomes.

Discussion

Patients with bullous diseases present unique anesthetic challenges due to widespread mucocutaneous fragility and multisystem involvement. This review demonstrates that most perioperative precautions described in the literature focus on mechanical and thermal protection of the skin and mucosa, highlighting how dermatologic insight can shape anesthetic safety. Across 26 studies, the most frequently reported interventions, such as padding for oximeters, non-adhesive ECG leads, lubrication of ophthalmologic and airway devices, and use of "egg-crate" mattresses, reflect the overarching principle of minimizing shear, pressure, and adhesive trauma.

Integumentary System

Skin fragility remains the most critical determinant of anesthetic technique. Use of non-adhesive monitoring devices, generous lubrication of the face and mask (using cortisol ointment or another lubricant), and gentle handling were universally recommended [16]. Postoperative reassessment of skin integrity and coordination with dermatology for wound care were also emphasized in order to prevent disease flare-ups and minimize the risk of skin infections that can evolve into systemic infections and other morbid complications.

Respiratory System

There have been previous suggestions that have suggested weak links between bullous diseases and comorbidities such as obstructive sleep apnea and asthma [37,38].

With regard to airway management, upper airway instrumentation should be minimized because the squamous epithelium lining the oropharynx and esophagus is very susceptible to trauma. Frictional trauma to the oropharynx, such as that produced by an oral airway, can result in the formation of large intraoral bullae and/or extensive hemorrhage from denuded mucosa. Nasal airways are equally hazardous. Esophageal stethoscopes should be avoided. Hemorrhage from ruptured oral bullae has been treated successfully by application of epinephrine-soaked gauze directly to the bullae. Furthermore, the literature supports fiberoptic or video-assisted intubation, with generous lubrication of the laryngoscope blade with cortisol ointment and/or petroleum jelly, and the selection of a smaller-than-usual endotracheal tube [16]. Humidified oxygen and tape-free fixation methods are recommended intraoperatively.

Great care must be taken to avoid trauma to the airway when intubating. Multiple attempts can lead to airway edema and prolonged intubation. Chronic inflammatory changes in the trachea lead to vocal cord narrowing and subglottic stenosis.

Following intubation, the tube must be carefully immobilized with soft cloth bandages to prevent movement in the oropharynx, and the tube must be positioned so that it does not exert lateral forces at the corners of the mouth. Tape should not be used to hold the endotracheal tube in place. May need to consider having surgical backup in the room if there is pronounced subglottic stenosis and concern for emergent cricothyroidotomy.

Cardiovascular and Metabolic Considerations

Systemic inflammation and chronic corticosteroid therapy contribute to hypertension, dyslipidemia, and cardiovascular disease [4,6]. Preoperative optimization and maintenance of hemodynamic stability are key. Stress-dose corticosteroids should be administered intraoperatively, and glucose, blood cell count, and electrolyte levels should be closely monitored [39]. Chronic hypertension with poor control can lead to various cardiac abnormalities, including left ventricular hyperplasia or even aortic dissection. A preoperative EKG should be done, and an echo can be considered to rule out cardiac manifestations.

Neurologic and Musculoskeletal Considerations

Patients with neurologic comorbidities may have altered airway reflexes or reduced cooperation. Sedation and opioids should be minimized to reduce delirium and pruritus risk, and multimodal analgesia should be favored [29,40]. Positioning precautions are critical for individuals with contractures or osteoporosis, with ample padding and avoidance of shear forces [16]. There have been associations with stroke, Parkinson’s, and dementia. Anesthetic delivery to minimize postoperative cognitive decline is needed.

Propofol and ketamine are useful for avoiding airway manipulation when the operative procedure does not require controlled ventilation or skeletal muscle relaxation. Despite the presence of dystrophic skeletal muscle, there is no evidence that these patients are at increased risk of a hyperkalemic response when treated with succinylcholine. There are no known contraindications to the use of volatile anesthetics in these patients. As alternatives to general anesthesia, regional anesthetic techniques (spinal, epidural, and brachial plexus block) have been recommended.

Although controversial, the use of entropy and BIS monitors can be useful to administer a sufficient dose of anesthesia. Pain control can be especially difficult for patients with bullous diseases. Multimodal pain management is recommended to decrease overall opioid consumption. Opioids have been found to cause pruritus, which exacerbates bullous crises. Although there is no absolute contraindication to regional or neuraxial anesthesia, the introduction of a needle into an active bullous site is not recommended, as it may necessitate general anesthesia and additional medications, including ketamine, precedex, NSAIDs (nonsteroidal anti-inflammatory drugs), and others. Opioids can be given but should be given with caution so as not to exacerbate a flare and worsen the current condition.

Renal, Hematologic, and Immune Considerations

Renal impairment from disease activity or drug toxicity necessitates dose adjustments for renally cleared anesthetics [1,41,42]. Immunosuppressive therapy and chronic corticosteroid use warrant preoperative CBC, coagulation studies, and strict aseptic technique [16,43]. Non-adhesive dressings and vigilant postoperative monitoring for infection or bleeding are advised. Drug interactions must be considered preoperatively to avoid intraoperative or postoperative complications.

Infection prevention is of paramount concern when completing regional and neuraxial anesthesia. Skin infections in patients with bullous disease can quickly spread to systemic infections. This is a concern for all surgical patients but can be especially risky in patients undergoing cardiac, neurological, and joint replacement surgery.

Psychiatric Considerations

Underlying psychiatric disorders can impact perioperative cooperation and recovery. Continuation of psychoactive medications and preoperative counseling may help minimize stress responses and delirium [43]. Drug interactions must be considered in continuing medications the day of surgery versus holding preoperatively.

## Conclusions

Collectively, these findings underscore that while the anesthetic management of bullous disease has traditionally centered on protecting fragile skin, comprehensive care must also address systemic and pharmacologic factors that influence intraoperative and postoperative outcomes. Although current evidence is limited to case reports and small series, two consistent themes have emerged: meticulous avoidance of friction and adhesives and careful airway planning. Yet, gaps remain regarding the selection of induction and analgesic agents, management of immunosuppressive therapies, and long-term postoperative outcomes. Future larger, prospective studies are needed to establish standardized protocols and better define the risk-benefit considerations of anesthetic techniques in this rare but challenging patient population.
